# Could Proprioceptive Stimuli Change Saddle Pressure on Male Cyclists during Different Hand Positions? An Exploratory Study of the Effect of the Equistasi^®^ Device

**DOI:** 10.3390/sports10060088

**Published:** 2022-06-02

**Authors:** Annamaria Guiotto, Fabiola Spolaor, Giovanni Albani, Zimi Sawacha

**Affiliations:** 1 Department of Information Engineering, University of Padova, 35131 Padova, Italy; annamaria.guiotto@unipd.it (A.G.); fabiola.spolaor@unipd.it (F.S.); 2 Istituto Auxologico Italiano, IRCCS, 28921 Verbania, Italy; g.albani@auxologico.it; 3 Department of Medicine, University of Padova, 35128 Padova, Italy

**Keywords:** pressure saddle, cycling posture, hand position, proprioception, Equistasi^®^ device

## Abstract

When pedaling, the excessive pressure on the seat has the potential to produce injuries and this can strongly affect sport performance. Recently, a large effort has been dedicated to the reduction of the pressure occurring at the saddle region. Our work aims to verify the possibility of modifying cyclists’ pedaling posture, and consequently the pressure on the saddle, by applying a proprioceptive stimulus. Equistasi^®^ (Equistasi srl, Milano, Italy) is a wearable device that emits focal mechanical vibrations able to transform the body temperature into mechanical vibratory energy via the embedded nanotechnology. The data acquired through a pressure mapping system (GebioMized^®^) on 70 cyclists, with and without Equistasi^®^, were analyzed. Pedaling in three positions was recorded on a spin trainer: with hands on the top, hands on the drop handlebar, and hands on the lever. Average force, contact surface, and average and maximum pressure each in different regions of the saddle were analyzed, as well as integral pressure time and center of pressure. In the comparisons between hands positions, overall pressure and force variables were significantly lower in the drop-handlebar position at the rear saddle (*p* < 0.03) and higher in hand-on-lever and drop-handlebar positions at the front saddle (*p* < 0.01). When applying the Equistasi device, the contact surface was significantly larger in all hand positions (*p* < 0.05), suggesting that focal stimulation of the lumbar proprioceptive system can change cyclists’ posture.

## 1. Introduction

Cycling has been recognized as an important means of promoting public health and it is among the most popular sports [1,2,3]. However, the constant pressure exerted on the bicycle seat might be the cause of non-traumatic injuries [4], ranging from saddle sores to more serious disorders related to the urogenital system [5]. 

In the last few years, the scientific literature focused on the overload injuries affecting the genitourinary tract because of their strong impact on both cyclists’ quality of life and sport performance: the mechanical causes of these injuries and the countermeasures applied to reduce the problem represent the objective of most of the studies [6]. A cumulative incidence of erectile dysfunction has been reported [7] and in particular, the most common urogenital problems, associated with cycling, are nerve entrapment syndromes associated with genital numbness, which is reported in 50–91% of cyclists, followed by erectile dysfunction reported in 13% to 24%. The most likely cause of both genital numbness and erectile dysfunction appears to be compression and elongation of the pudendal nerve near critical points such as the ischial tuberosity and the pubic arch during pedaling [6]. Taking into account that excessive pressure leads to transient hypoxemia of the nerve, the duration of these compressions seems to be more relevant than the intensity of the pressure itself [7,8]. Mechanical compression of the perineal tissue has also been studied by tomography [9] and magnetic resonance imaging [10] and has identified the lower region of the pubic symphysis as the most compressed part. Reducing the compressive load on the corresponding soft tissues has become the main goal of the evolution of bicycle saddle geometries [11,12,13]. In this context, it has been shown that saddle design can be defined by taking into account the influence of sex, power, hand position, and ischial tuberosities on saddle pressure during stationary cycling [11,12,13]; for instance, a partial nose saddle design resulted in a more comfortable seat than a standard or complete saddle without the nose [14]. Along with the development of more ergonomic saddles, it has been shown that the adoption of a correctly fitted bike can prevent many of the overuse injuries that occur from poor position. Among bike fitting operations, the measure of saddle pressure can provide an assessment of the stability of the cyclist’s position [15]. Hence, one of the main challenges is becoming the personalization of bike posture, which combines several factors such as age, style of riding, physical attributes such as flexibility, and anatomical variants [16,17].

Differently from the majority of the work that focused on improving athletes posture starting from changing bicycle saddle geometries or developing specific equipment, we aimed at verifying if the stimulus of the somatosensory system could change the athlete position on the saddle during cycling, thus modifying the saddle pressures. 

Equistasi^®^ is a neurorehabilitation device based on focal mechanical vibrations [18,19]. Muscle spindle endings transmit proprioceptive signals to the central nervous system that modulate the spinal reflexes excitability or the muscle responses elicited by postural perturbations. Previous studies documented that the higher motor centers increase the proprioceptive information that underlies motor control if stimulated by the vibrations. Once applied on the affected muscle areas, Equistasi^®^ interacts with the mechanoreceptors, the Golgi tendon organs, and the neuromuscular spindles. In this context, alternate vibratory stimulation on trunk muscles were shown to provide an improvement of trunk sway [20] or gait [21] in Parkinson’s disease individuals. In particular, beneficial effects on trunk stability were found in those stimulated with a wearable postural stabilizer, which provided prolonged muscle mechanical vibrations [20]. For these reasons, the Equistasi^®^ device is commonly used to assist in the rehabilitation of neurological diseases to improve both gait and posture [22,23,24]. Based on these considerations, we can assume that applying prolonged muscle mechanical vibrations, by means of a wearable postural stabilizer, could provide the proprioceptive information necessary to improve motor control that underlies postural stability. 

Considering that many of the overload injuries that affect cyclists occur from poor posture, we hypothesized that we could change the position of the athletes on the bicycle, by providing a vibratory stimulus through Equistasi^®^. The purpose of this study was therefore to verify the effect of the device in inducing efficient postural changes during pedaling, by measuring the pressure on the saddle.

## 2. Materials and Methods

### 2.1. Subjects

**Figure 1:**
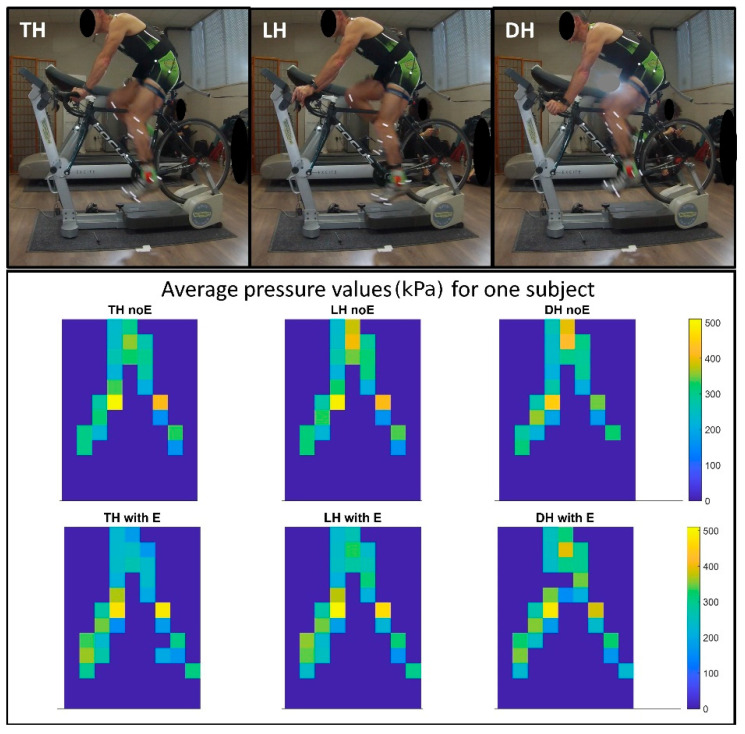
Acquisition setup and example of saddle pressures for one subject in the top handlebar (TH), hands on lever (LH), and drop handlebar (DH) positions without Equistasi^®^ (noE) and with Equistasi^®^ (E).

This study was a secondary analysis of an existing database that included data from 70 amateur cyclists, all men (mean ± standard deviation age 33.41 ± 13.5 years and BMI 21.53 ± 2.44 kg/m^2^), acquired while pedaling on a spin trainer between May 2011 and December 2019. The data were retrospectively extracted from the database available at BiomovLab (University of Padua), after appropriate anonymization. The inclusion criteria were: age of the participants >16 years [25] (upper limits of pediatric age according to the Italian guidelines), the participants must have traveled at least 6/9000 km per year, available tests in the database corresponding to 3 hand positions: top handlebar, hands on the lever, and hands on the drop handlebar (hereafter referred to as TH, LH, and DH, respectively) (Figure 1), signed informed consent available to participate in the data collection. The exclusion criteria were: age < 16 years (i.e., pediatric age), and documented presence of pathology of the nervous system or cardiac pacemaker, diabetes and orthostatic hypotension, and absence of consent to participate in the data collection. All participants were amateur cyclists included in the categories from Master 1 to Master 3 levels according to the Italian Cycling Federation [26].

### 2.2. Acquisition Protocol

According to the configuration already proposed in other studies [22], the device was applied on the skin as follows: one on the seventh cervical vertebra and two on each soleus muscle. This previously adopted configuration determined the changes in the center of pressure (COP) induced by the vibration of the leg and the paraspinal muscles [21,27,28]. After a 10-min warm-up, subjects were asked to perform 10-min cycling sessions at 180 Watts, in three different hand positions: TH, DH, and LH. 

Athletes were tested at the same work rate of 180 Watts as it was considered an average intensity for our population of subjects, similarly to [16,29,30,31] who chose to test their subjects at a fixed work rate. Each athlete could then choose the preferred cadence that allows him to reach the 180 W.

Each session lasted 40 mins with 4 intervals of 10 mins: warm up, cycling with TH, cycling with LH, and cycling with DH. 

A physiotherapist was in charge of applying the Equistasi^®^ device.

### 2.3. Instrumental Protocol

The bike, positioned on the spin trainer, was instrumented with a wireless saddle pressure mapping system (GebioMized^®^) consisting of a thin flexible mat with 64 piezoresistive sensors (1.6 mm thick), fitted over the saddle, which acquired the data (200 Hz) and sent them to the computer. The sensors were arranged in 9 columns and 12 rows on a sensorized surface of 32.154 cm^2^ and each circular sensor had a diameter of 8 mm with an inter-sensor distance of 7 mm longitudinally and 6 mm in the medial lateral direction. The instrument was calibrated by the manufacturer. Each sensor’s resistance was a function of forces normal to the surface with a recording range between 50 and 300 kPa.

The dynamic contact pressure maps were recorded during the last 10 s of each hand position within each session [12,32]. The variables extracted from the contact pressures maps for the whole saddle (WS), the anterior part of the saddle (AS, corresponding to the pubic bone area), and the posterior part of the saddle (PS) by means of self-developed algorithms (Matlab R2019b) are reported in Table 1.

All the pressure and force measurements were reported in percentage of the body weight (BW).

### 2.4. Equistasi^®^ Device

Equistasi^®^ is a wearable medical device (1×2 cm in size and has a feather-light weight of 0.17 g), approved by the Ministry of Health on 05-08-2010 with CNN product code number 342575 and COP product code number 342577. It is based on vibrational technology: it self-generates focal mechanical vibrations at a non-constant frequency of about 9000 Hz, within the limits imposed by Legislative Decree 81/08. Subjects do not feel the focal mechanical vibrations, they feel just the band aid (Supplementary Material, part A, Figure S1).

### 2.5. Statistical Analysis

After checking for normality (Lilliefors test), the two-way ANOVA or the non-parametric Friedman test were used where appropriate, to compare the data grouped according to the condition with and without Equistasi^®^ device or the hand positions (X2 or F values and *p*-values were reported in Supplementary Material, part B, Tables S1 and S2). Significance was accepted with *p*-value <0.05 (Bonferroni correction was applied when necessary). As post-hoc, Student paired *t*-test or Wilcoxon signed rank test were used where appropriate, for the comparisons among conditions or among hand positions (Z or T values and *p*-values were reported in Supplementary Material, part B, Tables S3 and S4). The statistical analyses were performed using Matlab (R2019b).

## 3. Results

**Figure 2:**
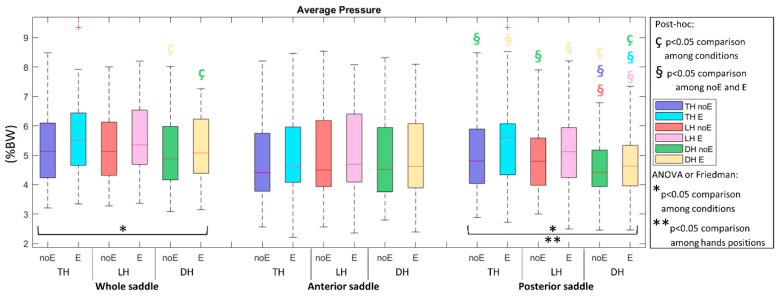
Average pressure in the whole saddle, anterior saddle, and posterior saddle when cycling in TH, LH, and DH positions, in the conditions without (noE) and with Equistasi^®^ (E). * and ** mean a statistically significant differences (*p* < 0.05) with 2 way-ANOVA or Friedman test in the comparison among conditions (*) or hands positions (**). ç and § mean a statistically significant difference (*p* < 0.05) with post-hoc test in the comparison among conditions (ç) or hands positions (§): blue with respect to TH noE, light blue with respect to TH E, red with respect to LH noE, pink with respect to LH E, green with respect to DH noE, yellow with respect to DH E.

**Figure 3:**
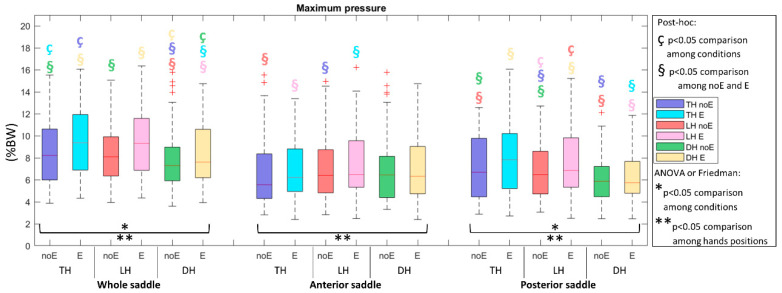
Peak pressure in the whole saddle, anterior saddle, and posterior saddle when cycling in TH, LH, and DH positions, in the conditions without (noE) and with Equistasi^®^ (E). * and ** mean a statistically significant differences (*p* < 0.05) with 2 way-ANOVA or Friedman test in the comparison among conditions (*) or hands positions (**). ç and § mean a statistically significant difference (*p* < 0.05) with post-hoc test in the comparison among conditions (ç) or hands positions (§): blue with respect to TH noE, light blue with respect to TH E, red with respect to LH noE, pink with respect to LH E, green with respect to DH noE, yellow with respect to DH E.

**Figure 4:**
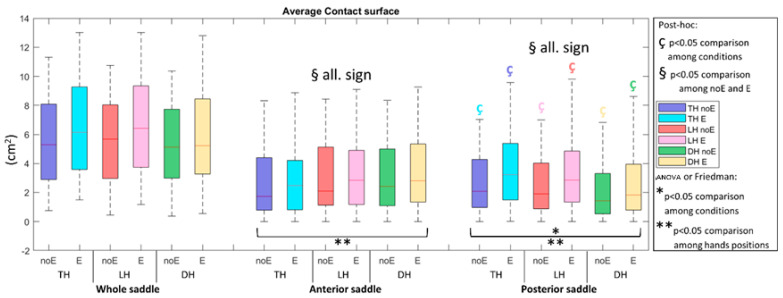
Average contact surface in the whole saddle, anterior saddle, and posterior saddle when cycling in TH, LH, and DH positions, in the conditions without (noE) and with Equistasi^®^ (E). * and ** mean a statistically significant differences (*p* < 0.05) with 2 way-ANOVA or Friedman test in the comparison among conditions (*) or hands positions (**). ç and § mean a statistically significant difference (*p* < 0.05) with post-hoc test in the comparison among conditions (ç) or hands positions (§): blue with respect to TH noE, light blue with respect to TH E, red with respect to LH noE, pink with respect to LH E, green with respect to DH noE, yellow with respect to DH E.

**Figure 5:**
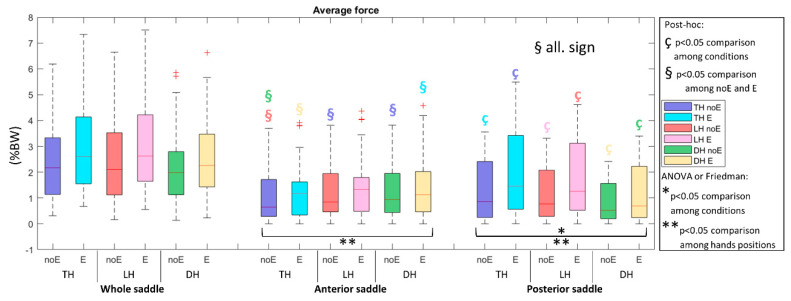
Average force in the whole saddle, anterior saddle, and posterior saddle when cycling in TH, LH, and DH positions, in the conditions without (noE) and with Equistasi^®^ (E). * and ** mean a statistically significant differences (*p* < 0.05) with 2 way-ANOVA or Friedman test in the comparison among conditions (*) or hands positions (**). ç and § mean a statistically significant difference (*p* < 0.05) with post-hoc test in the comparison among conditions (ç) or hands positions (§): blue with respect to TH noE, light blue with respect to TH E, red with respect to LH noE, pink with respect to LH E, green with respect to DH noE, yellow with respect to DH E.

**Figure 6:**
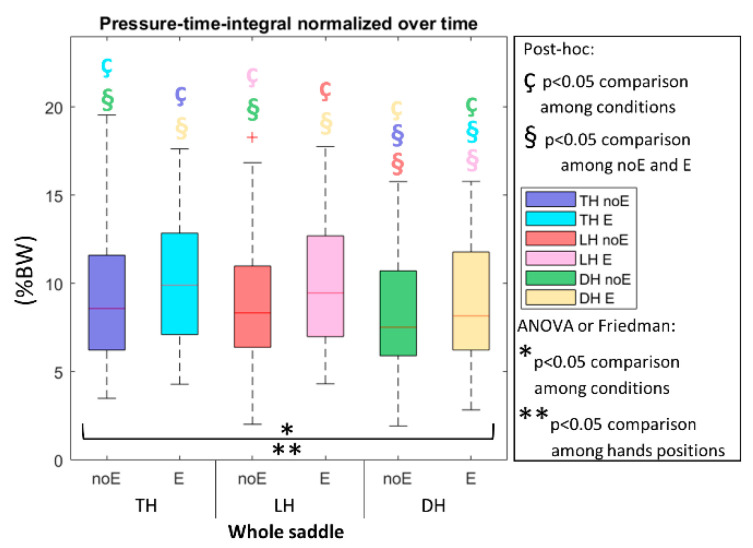
Pressure-time-integral normalized on the duration of the complete acquisition on the whole saddle, when cycling in TH, LH, and DH positions, in the conditions without (noE) and with Equistasi^®^ (E). * and ** means a statistically significant differences (*p* < 0.05) with 2 way-ANOVA or Friedman test in the comparison among conditions (*) or hands positions (**). ç and § mean a statistically significant difference (*p* < 0.05) with post-hoc test in the comparison among conditions (ç) or hands positions (§): blue with respect to TH noE, light blue with respect to TH E, red with respect to LH noE, pink with respect to LH E, green with respect to DH noE, yellow with respect to DH E.

**Figure 7:**
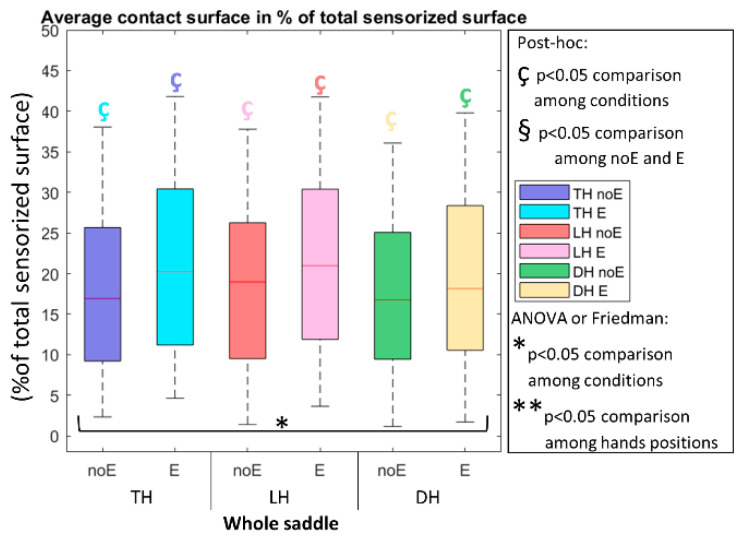
Average contact surface in percentage of the total sensorized surface on the whole saddle, when cycling in TH, LH, and DH positions, in the conditions without (noE) and with Equistasi^®^ (E). * means a statistically significant differences (*p* < 0.05) with 2 way-ANOVA or Friedman test in the comparison among conditions. ç means a statistically significant difference (*p* < 0.05) with post-hoc test in the comparison among conditions: blue with respect to TH noE, light blue with respect to TH E, red with respect to LH noE, pink with respect to LH E, green with respect to DH noE, yellow with respect to DH E.

**Figure 8:**
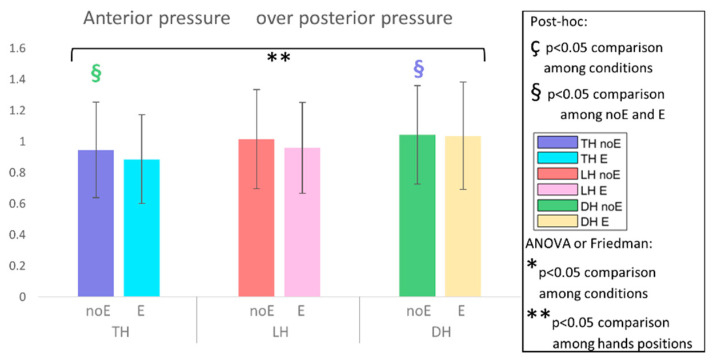
Ratio between the average pressure on the anterior saddle and posterior saddle, when cycling in TH, LH, and DH positions, in the conditions without (noE) and with Equistasi^®^ (E). ** means a statistically significant differences (*p* < 0.05) with 2 way-ANOVA or Friedman test in the comparison among hands positions. § means a statistically significant difference (*p* < 0.05) with post-hoc test in the comparison among hands positions: blue with respect to TH noE, light blue with respect to TH E, red with respect to LH noE, pink with respect to LH E, green with respect to DH noE, yellow with respect to DH E.

**Figure 9:**
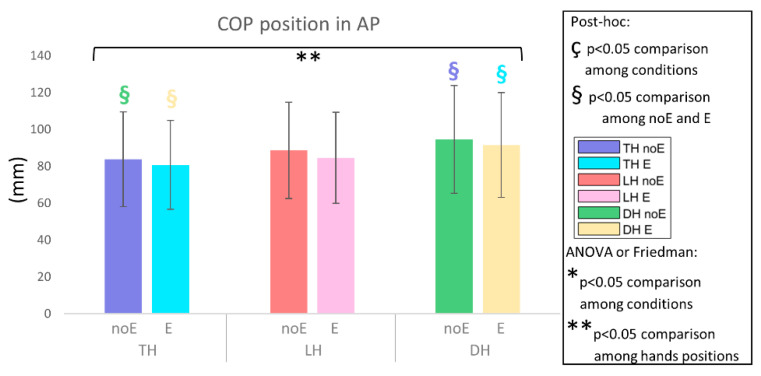
Position of the COP in the anterior-posterior direction on the whole saddle when cycling in TH, LH, and DH positions, in the conditions without (noE) and with Equistasi^®^ (E). ** means a statistically significant differences (*p* < 0.05) with 2 way-ANOVA or Friedman test in the comparison among hands positions. § means a statistically significant difference (*p* < 0.05) with post-hoc test in the comparison among hands positions: blue with respect to TH noE, light blue with respect to TH E, red with respect to LH noE, pink with respect to LH E, green with respect to DH noE, yellow with respect to DH E.

**Figure 10:**
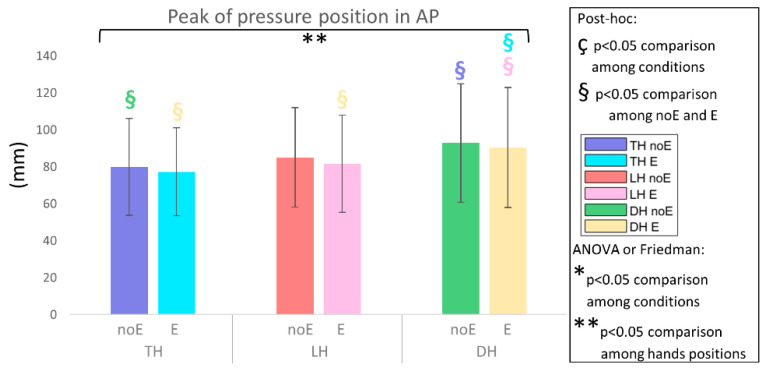
Position of the pressure peak in the anterior–posterior direction on the whole saddle, when cycling in TH, LH, and DH positions, in the conditions without (noE) and with Equistasi^®^ (E). ** means a statistically significant differences (*p* < 0.05) with 2 way-ANOVA or Friedman test in the comparison among hands positions. § means a statistically significant difference (*p* < 0.05) with post-hoc test in the comparison among hands positions: blue with respect to TH noE, light blue with respect to TH E, red with respect to LH noE, pink with respect to LH E, green with respect to DH noE, yellow with respect to DH E.

Pedaling cadence over the 70 subjects was 95.4 ± 9.5 rpm (mean ± standard deviation), with no significant differences among the hand positions or the presence/absence of Equistasi. Results concerning the pressure variables were reported in the following figures and tables (Figure 2, Figure 3, Figure 4, Figure 5, Figure 6, Figure 7, Figure 8, Figure 9 and Figure 10, Table 2, and Tables S1–S9 and the Figures S2 and S3 in the Supplementary Materials, part B and C).

During the acquisition with the Equistasi^®^ device, a statistically significant increment was observed in both average and peak pressure. Specifically, pressure_E in the DH position (*p* = 0.0176 and *p* = 0.0113, respectively, in the WS and PS, Figure 2) and peak pressure_E in all the recorded positions (*p* < 0.01) in TH and DH in the WS and *p* < 0.001 in LH in the PS, Figure 3).

For what concerns the contact surface with Equistasi^®^ device (surface_E), a statistically significant increment was observed in the PS (*p* < 0.001) in all the hand positions (Figure 4).

Regarding the force in the condition with Equistasi^®^ device (force_E), a statistically significant increment was noticed in all positions in the PS (*p* < 0.015) (Figure 5).

When considering the pressure-time-integral_E and the percentage surface_E, significantly higher values were detected in the condition with Equistasi^®^ (Figure 6 and Figure 7, respectively).

In the analysis of the pressure ratio_E, the peak position_E, and the COP_E no significant differences were detected (Figure 8, Figure 9 and Figure 10).

Regarding the results of the comparison among the hand positions while cycling (Table 2), the following statistically significant differences were observed: in the comparison between TH and LH positions, no statistically significant differences were noticed on the WS, neither without Equistasi^®^, nor with Equistasi^®^; a different situation was observed on the AS where a significant increase (*p* < 0.005) of each variable in the LH position was noticed in both conditions, with the only exception of the force_E and the average pressure. In the PS, a significant decrease in the pressure peak, contact surface, and average force (*p* < 0.03) was displayed in the LH position in both conditions, with the only exception of the peak pressure_E. 

In the comparison between TH and DH positions, on the WS, a statistically significant decrease (*p* < 0.003) was observed on the pressure peak in the DH position in both conditions; while an opposite trend was highlighted on the pressure ratio_noE that significantly (*p* = 0.014) increased. With respect to the force and the pressure ratio, no significant differences were noticed in the condition with the Equistasi^®^ device. A significant anterior shift in both the COP and peak position was detected during the DH position in both conditions (*p* < 0.027). In correspondence to the AS, the surface, the force, the force in correspondence to the pressure peak, and the surface in correspondence to the pressure peak significantly increased (*p* < 0.001) in both conditions, with the exception of the force in peak_E. When considering the PS, all the variables significantly decreased (*p* < 0.022) in the DH position in both conditions, with the only exception of the force in peak_E.

Finally in the comparison between the two hand positions LH and DH on the WS, the pressure peak significantly decreased in the DH position in both conditions (*p* < 0.001). When considering the AS, a significant increase was detected only on the contact surface in both conditions in the DH position (*p* < 0.025). Meanwhile, on the PS, a significant decrease (*p* < 0.024) was recorded on all the variables in the DH position, with the only exception of the force in peak_E.

## 4. Discussion

Based on our hypothesis, this study focused on the possibility to change cyclists’ sitting posture by providing stimuli to the proprioceptive system. To this end, the effect of the Equistasi^®^ device was tested, based on the results of Alfonsi et al. 2015, who documented its influence on the mechanisms involved in the physiological control of movement and posture [19].

It should be noticed that, from a biomechanical point of view, a new aspect of our contribution could be demonstrated in the assessment of the athletes in LH position, which to the best of our knowledge, was not previously documented. 

Our results found statistically significant differences through the use of Equistasi^®^ in distributing the pressure over a larger contact surface associated with a shift from the anterior to the posterior region of the saddle rather than a peak pressure reduction. This was achieved in association with a greater force in the PS recorded when the Equistasi^®^ device was applied. Although our results showed an increase in saddle forces associated with the application of the Equistasi^®^ device, this could be interpreted as a change in seat posture. This finds agreement with previous literature [33,34,35,36], that detected an increment in saddle forces in association with a reduction in hip vertical reaction forces with a consequent reduction in pedal reaction forces, and an upward acceleration of the trunk. Nevertheless, the increment recorded in the posterior contact surface could be associated with a forward pelvic tilt that has been reported to promote a decrease of lumbar flexion and tensile stress to the longitudinal ligaments of the lumbar spine [36]. This change in posture could help in reducing the incidence of low back pain, along with distributing a greater percentage of the body weight over the handlebars, thus promoting a reduction of the load placed on the lumbar vertebrae [36]. However, future studies including the assessment of the load distribution over the handlebars together with trunk and pelvis kinematics are needed to confirm this hypothesis. 

In the attempt to compare our results with the state of the art in terms of forces exerted on the saddle, to the best of our knowledge, only four studies have measured saddle forces in cycling [33,34,35,36] and the results appear to be contradictory. Three studies investigated the association between saddle forces and cadence, two identified a direct relationship between maximum magnitudes of the force decreasing along with cadence [33,35] and one described an opposite trend [34]. Another study observed that the vertical reaction force of the saddle decreases mainly in response to an increase in the hip vertical reaction force; consequently, with increasing pedal reaction forces, the support provided by the saddle to body weight decreases [36]. Considering that, according to Newton’s second law, the force exerted on the saddle is the result of a mechanical interaction between the cyclist’s body weight and the other forces applied on his bicycle, we can hypothesize that the increase in saddle forces recorded in our study was the result of a reduction in hip vertical reaction force, which previous literature documented in association with a decrease in pedal reaction forces [36].

Of course, this requires further research including the evaluation of other forces acting on the rider such as the pedal reaction forces, trunk, and handlebar forces [36]. However, we could still speculate that this specific change in cycling posture is related to changes in the trunk’s center of mass acceleration, which should be complementary to the saddle force. Based on the results of Costes et al. 2015, when greater forces are applied to the saddle, the trunk center of mass accelerates upward [36]. Conversely, when lower saddle forces are generated, the trunk center of mass accelerates downward [36]. The increase in saddle forces documented when the Equistasi^®^ device was applied seems to suggest that the device promoted an upward acceleration of the trunk center of mass. This is in agreement with the overall increase observed in the contact area which increased in the PS. To confirm this hypothesis, future studies should consider measuring trunk acceleration. 

With respect to the results on the mean pressure measures, our data are generally higher than those reported in the literature [12,37]. For instance, our study documented mean values for the anterior and posterior pressures in the top handlebar position ranging between 32.04 and 35.80 kPa, while the mean anterior and posterior pressures recorded in the study of Schrader et al. 2002 ranged between 15.9 and 20.0 kPa [37]. Similar values were also reported in Bressel and Cronin 2005 [12] ranging between 11.2 and 21.1 kPa. An opposite situation can be observed in the pressure peak that resulted lower in our study, ranging between 39.5 and 54.4 kPa in comparison with the above-mentioned studies that reported values ranging between 59.9 and 145.1 kPa [12]. 

The differences between our results and what is reported in the literature might be due to the different approach adopted. First, in our study, pressure measurements were recorded for 10 s at an external work rate of 180 Watts positioned on top of their own seat, meanwhile in Bressel and Cronin 2005, pressure measures were recorded for 5 s at an external work rate of 118 and 300 Watt and a unisex seat was used for all the subjects [12]. Differences can also be seen on the pressure mat used in the study, which in our case was composed of 64 sensors that acquired the pressure distribution with a measurement frequency of 200 Hz, while in Bressel et al. 2005, a system composed by 768 piezoresistive sensors with a measurement frequency of 5 Hz was adopted. Unlike our study and Bressel and Cronin 2005 [12], in Schrader et al. 2002, the pressure exerted between the participant and the bicycle saddle was measured with a thin profile resistance-based pressure measurement mat with 32 sensors at 5 Hz, and each participant was tested on two different seats (e.g., split saddle design, W-groove saddle design) [37].

Considering the comparison between the pressure distribution without and with the Equistasi^®^ device, our results showed statistically significant differences in the mean pressure that increased in the DH position on the WS and PS. It is worth noticing that our athletes in both acquisition settings did not show a significant difference in the ratio of anterior to posterior pressure in each the tested position (i.e., DH, LH, TH). These results indicate that, although a significant increase in specific seat regions was documented, the Equistasi^®^ device promoted a shift from the AS region to the PS region in the DH position. This change in saddle pressure reflects a change in seat posture that is confirmed by the results of the COP displacement. Indeed, a shift from the anterior to the posterior region of the saddle causes a reduction in the pressure exerted under the pubic area. Unfortunately, our results were only able to document a shift of the load from the anterior to the posterior region of the saddle without showing a statistically significant reduction in the pressure peak. Future studies are needed in order to verify the possibility to induce this change through the Equistasi^®^ device.

When taking into account the differences across the 3 hand positions, it should be noticed that a decrease in the ratio between the pressure applied on the AS and PS was observed when moving from the DH position to the TH position, thus documenting a shift of the pressure from the AS to the PS. This finding is in agreement with the results of Bressel and Cronin 2005 [12], who reported greater pressure values in the PS during the TH position when compared to the DH. In terms of pressure peak, the values were significantly greater in the WS during the TH position compared with the DH one. In the PS only, the TH position showed greater values than the DH position, similarly to what was described in Bressel and Cronin 2005 [12]. This behavior can be attributed to the different posture that characterizes the TH position with respect to the DH, where pelvic and trunk angles are generally lower, accompanied by a lower activity of triceps brachii [38]. It should be further mentioned that in the LH position, an increase on all the variables relative to the AS, with respect to the TH, was documented, while in the PS, lower values were recorded with respect to the TH position and higher than the DH. From a prevention point of view, our results showed that the DH position presented lower values in all the analyzed variables associated with an anterior shift of the COP, when compared with other hand positions. This result found agreement with the work of Slane et al. 2011 [39], who demonstrated that in the DH position a higher peak pressure was detected on the wrist when compared with other positions, thus concluding that during DH the saddle is unloaded in favor of a larger load on the hands. This brings to the conclusion that the prevention strategies should be a compromise between reducing load over at risk anatomical structure without producing excessive loads on other structures that might become at risk. On the side of our results associated with the use of mechanical vibration to induce a change in posture while cycling, we can conclude that a shift in the weight was observed from the anterior to the posterior saddle, but this was not accompanied by a significant reduction in the saddle pressure. The lack of significant changes could be associated to the short time monitored through the pressure saddle; hence, future development might include recording the pressure over the whole task duration (10 min vs. 10 s). Overall, in addition to the statistically significant results, the study suffers from important limitations that should be acknowledged. First of all, in this study, we lack a long-term effect, as the measurements were performed only after the immediate application of the Equistasi^®^ device; this is potentially a relevant limitation if we consider that in previous studies, that involved pathological subjects, the efficacy of the device was reported after 4 weeks of treatment [22,23,24]. In this study, however, we focused on evaluating the immediate effect of the device, also based on what was reported by the previous neurophysiological studies [19], that concluded that Equistasi^®^ has a modulatory effect on the proprioceptive reflex circuits. Indeed, in the former study, high-frequency microvibrations significantly increased the inhibitory effect of TVS on the H reflex for up to three minutes. Secondly, the six different conditions (TH, DH, LH * with/without Equistasi^®^) were always tested in the same order, similarly to Bressel and Cronin 2005 [12]. Even if a similar choice was made by Bressel et al. [38] who studied the influence of bicycle seat design on cyclist posture, it represents one of the limitations of our study. Another limitation can be found in the differences existing among cyclists’ saddles that might have affected the different responses documented among the athletes. Differently from other studies that adopted the same saddle across all participants [12,40], in our study, each participant was tested on their own saddle. However, our choice was based on the consideration that a change in the saddle might have limited the response to a stimulation of the proprioceptive system (i.e., Equistasi^®^), given the need of the body to adapt to an unusual seat. When we consider the sites of application of the Equistasi^®^ device (setup), the gluteal muscles and the trunk muscles were not taken into account: this choice was driven by the results of previous studies that investigated the role of Equistasi^®^ in promoting a better postural control [21,22,27,28]. Future studies could explore different positions, including stimulation of the glutei as well as the trunk and legs muscles. Among the various limitations, the recording time should be included as only 10 s of saddle pressure were acquired out of a 10 min test. This could have prevented us from documenting further changes; future studies acquiring the saddle pressure over a longer period of time are therefore needed. With regard to the choice of acquiring the athletes only at one work rate, this prevented us from verifying the effect of the device on different cadences; however, this was out of the scope of the present study. Nevertheless, only males were measured in this study, and, therefore, the results cannot be transferred to females. Finally, participants were not blinded to the Equistasi^®^ conditions: due to the fact that the device self-generates focal mechanical vibrations at a non-constant frequency of about 9000 Hz, not perceptible from tactile receptors, we recognize that a placebo device should have been provided to each participant, similarly to what was previously done by the authors in the Parkinson’s disease study [22,23,24]. However, it should be mentioned that this study was a secondary analysis of an existing database.

Regardless of the important limitations, our preliminary results could be used in order to plan a larger study design that avoids possible bias by acquiring both males and females, and by randomizing both the sequence of hand positions and the application of the device and/or placebo. Future developments could involve planning longer acquisition sessions in terms of pressure sensors recording, complemented by kinematics analysis and electromyography. This could allow providing a more complete analysis of the impact of proprioceptive stimuli on the overall cyclists’ biomechanics.

## 5. Conclusions

Our results showed that cyclists’ seat posture could be changed by stimulating the proprioceptive system of the athletes while pedaling. Overall, this change can be observed mainly in a redistribution of the pressure on a larger surface of the seat rather than on the reduction of the amount of pressure released on the saddle. 

When considering the overall analysis provided by our study, two main innovative aspects should be highlighted: first of all, several variables were newly introduced in this context, such as pressure time integral in the WS, contact surface in percentage of the sensorized surface in the WS, ratio between the average pressure on the AS and the PS part of the saddle, position of the pressure peak in the anterior–posterior direction in the WS, and position of the COP in the anterior–posterior direction in the WS. These variables are characteristic of pressure analysis in other domains such as gait analysis [41,42], but were newly introduced in the context of cycle biomechanics. Finally, for the first time the LH position, which is currently adopted by cyclists, was analyzed and revealed results that are different from the other two positions, thus supporting the need to be investigated separately. Future studies are needed in order to increase the sample of subjects, to evaluate the effect of applying proprioceptive stimuli either on a wider timeframe or at different work rates, and to analyze saddle pressure over the whole test duration. The adoption of a placebo device should also be explored in order to avoid possible bias related to the perceived presence of the Equistasi^®^ device.

## Figures and Tables

**Table 1:** List of variables calculated from the pressure maps.

VARIABLE	NAME OF THE VARIABLE	SADDLE ZONE	DEFINTION	CONDITION
average pressure	average pressure_noE and average pressure_E	WS, AS and PS	the average over all the time steps	without and with Equistasi^®^
peak pressure	peak pressure_noE and peak pressure_E	WS, AS and PS	the maximum pressure averaged over all the time steps	without and with Equistasi^®^
contact surface	surface_noE and surface_E	WS, AS and PS	sum of the sensors on, averaged over all the time steps	without and with Equistasi^®^
average force	force_noE and force_E	WS, AS and PS	“sensor pressure x sensor surface” and averaged over all the time steps	without and with Equistasi^®^
force in peak of pressure frame	force in peak_noE and force in peak_E	WS, AS and PS	force value corresponding to the frame of peak of pressure value	without and with Equistasi^®^
contact surface in peak of pressure frame	surface in peak_noE and surface in peak_E	WS, AS and PS	contact surface value in the frame of peak of pressure value	without and with Equistasi^®^
pressure-time-integral	pressure-time-integral_noE and pressure-time-integral_E	WS	area under the pressure-time curve over the entire acquisition, and normalized on the duration of the complete acquisition	without and with Equistasi^®^
contact surface in percentage	percentage surface_noE and percentage surface_E	WS	average of the contact surface in percentage of the total sensorized surface	without and with Equistasi^®^
ratio between AS and PS pressure	pressure ratio_noE and pressure ratio_E	WS	ratio between the average pressure on the AS and PS part of the saddle	without and with Equistasi^®^
antero-posterior position of the peak of pressure	peak position_noE and peak position_E	WS	position of the peak of pressure in the anterior-posterior direction, average over all the time steps	without and with Equistasi^®^
antero-posterior position of the COP	COP_noE and COP_E	WS	position of the COP in the anterior-posterior direction, average over all the time steps	without and with Equistasi^®^

**Table 2:** Summary of the statistically significant differences on the comparisons among the different hands positions (top handlebar—TH; hands on lever—LH; and drop handlebar—DH) in the whole saddle (WS), anterior saddle (AS), and posterior saddle (PS) for the conditions without and with the Equistasi^®^ device (noE and E, respectively). *p* values are reported in brackets where significant differences occur (paired *t*-test or Wilcoxon signed rank as post-hoc 2-way ANOVA or Friedman where appropriate). n.s. means no statistical significance differences among the comparisons.

WS	TH vs. LH		TH vs. DH		LH vs. DH	
	noE	E	noE	E	noE	E
average pressure	n.s.	n.s.	n.s.	n.s.	n.s.	n.s.
peak of pressure	n.s.	n.s.	lower in DH (0.003)	lower in DH (<0.001)	lower in DH (0.001)	lower in DH (<0.001)
contact surface	n.s.	n.s.	n.s.	n.s.	n.s.	n.s.
average force	n.s.	n.s.	n.s.	n.s.	n.s.	n.s.
Pressure-time-integral	n.s.	n.s.	lower in DH (<0.001)	lower in DH (<0.001)	lower in DH (<0.001)	n.s.
contact surface %	n.s.	n.s.	n.s.	higher in DH (0.014)	n.s.	n.s.
pressure ratio	n.s.	n.s.	n.s.	higher in DH (0.014)	n.s.	n.s.
peak position	n.s.	n.s.	anteriorized in DH (0.011)	anteriorized in DH (0.009)	n.s.	n.s.
COP position	n.s.	n.s.	anteriorized in DH (0.026)	anteriorized in DH (0.019)	n.s.	n.s.
**AS**	**TH** **vs. LH**		**TH** **vs. DH**		**LH** **vs. DH**	
	**noE**	**E**	**noE**		**noE**	**E**
average pressure	higher in LH (<0.001)	higher in LH (0.005)	n.s.	n.s.	n.s.	n.s.
peak of pressure	higher in LH (<0.001)	higher in LH (0.002)	n.s.	n.s.	n.s.	n.s.
contact surface	higher in LH (<0.001)	higher in LH (<0.001)	higher in DH (<0.001)	higher in DH (<0.001)	higher in DH (0.018)	higher in DH (0.025)
average force	higher in LH (<0.001)	n.s.	higher in DH (<0.001)	higher in DH (<0.001)	n.s.	n.s.
**PS**	**TH** **vs. LH**		**TH** **vs. DH**		**LH** **vs. DH**	
	**noE**	**E**	**noE**		**noE**	**E**
average pressure	n.s.	n.s.	lower in DH (<0.001)	lower in DH (0.002)	lower in DH (<0.001)	lower in DH (0.023)
peak of pressure	lower in LH (0.029)	n.s.	lower in DH (0.021)	lower in DH (0.001)	lower in DH (<0.001)	lower in DH (0.019)
contact surface	lower in LH (0.003)	n.s.	lower in DH (<0.001)	lower in DH (<0.001)	lower in DH (<0.001)	lower in DH (<0.001)
average force	lower in LH (0.002)	lower in LH (0.002)	lower in DH (<0.001)	lower in DH (<0.001)	lower in DH (<0.001)	lower in DH (<0.001)

## Data Availability

Data will be available upon specific request to the corresponding author.

## Supplementary Materials

The following supporting information can be downloaded at: https://www.mdpi.com/article/10.3390/sports10060088/s1, Supplementary material (part A, part B, and part C).
