# Supplementary material for: Could Proprioceptive Stimuli Change Saddle Pressure on Male Cyclists during Different Hand Positions? An Exploratory Study of the Effect of the Equistasi® Device
Source: Sports (Basel). 2022 Jun 2;10(6):88. doi: 10.3390/sports10060088 (PMC9227057; doi:10.3390/sports10060088)
Supplement: Supplementary file 1 [file sports-10-00088-s001.zip › sports-1682157-supplementary.pdf]

sports

MDPI

Article

Could Proprioceptive Stimuli Change Saddle Pressure on Male Cyclists during Different Hand Positions? An Exploratory Study of the Effect of the Equistasi® Device

Annamaria Guiotto [1,†], Fabiola Spolaor [1,†], Giovanni Albani [2] and Zimi Sawacha [1,3,*]

[1] Department of Information Engineering, University of Padova, 35131 Padova, Italy; annamaria.guiotto@unipd.it (A.G.); fabiola.spolaor@unipd.it (F.S.)
[2] Istituto Auxologico Italiano, IRCCS, 28921, Verbania, Italy; g.albani@auxologico.it
[3] Department of Medicine, University of Padova, 35128, Padova, Italy
* Correspondence: zimi.sawacha@unipd.it; Tel. +39-049-8277633
† These authors contributed equally to this work.

Supplementary material A

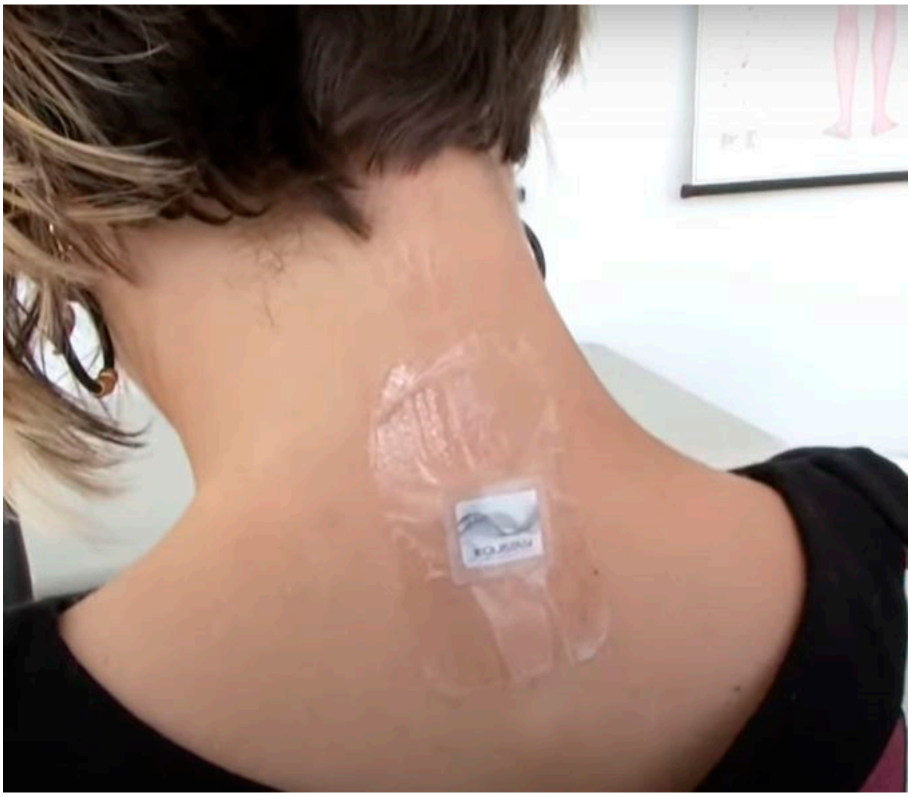

Figure S1. Example of band aid attachment for the Equistasi® device.

Supplementary material B

Table S1. Results of the statistical tests (2-way Anova or Friedman where appropriate) on the comparisons among the different hands positions without the Equistasi® device (top handlebar – TH – hands on lever – LH – and drop handlebar – DH) in the whole saddle (WS), anterior saddle (AS) and posterior saddle (PS).

X2 or F values	TH vs LH vs DH		
	WS	AS	PS
average pressure	4.2979	1.1295	15.9374
peak of pressure	6.4834	1.6268	17.2295
contact surface	1.8617	2.6913	10.0706
average force	3.4833	3.1219	11.8834
pti	6.3124	-	-
contact surface %	1.8731	-	-
pressure ratio	5.8237	-	-
peak position	7.2952	-	-
COP position	5.6217	-	-
**p values**	**TH vs LH vs DH**		
	WS	AS	PS
average pressure	0.1166	0.5685	0.0003
peak of pressure	0.0391	0.4433	0.0002
contact surface	0.3942	0.2604	0.0065
average force	0.1752	0.2099	0.0026
pti	0.0426	-	-
contact surface %	0.3920	-	-
pressure ratio	0.0033	-	-
peak position	0.0008	-	-
COP position	0.0039	-	-

Table S2. Results of the statistical tests (2-way Anova or Friedman where appropriate) on the comparisons among the different conditions with the Equistasi ® device for the hands positions (top handlebar – TH – hands on lever – LH – and drop handlebar – DH) in the whole saddle (WS), anterior saddle (AS) and posterior saddle (PS).

X2 or F values	TH vs LH vs DH		
	WS	AS	PS
average pressure	4.8814	2.2480	7.2247
peak of pressure	6.6186	1.6264	6.4517
contact surface	8.2195	0.9434	8.9615
average force	9.4248	1.7312	10.6238
pti	4.7966	-	-
contact surface %	8.7216	-	-
pressure ratio	0.2997	-	-
peak position	0.9932	-	-
COP position	1.6053	-	-
**p values**	**TH vs LH vs DH**		
	WS	AS	PS
average pressure	0.0271	0.1338	0.0072
peak of pressure	0.0101	0.2022	0.0111

**contact surface**	0.0041	0.3314	0.0028
**average force**	0.0021	0.1883	0.0011
**pti**	0.0285	-	-
**contact surface %**	0.0031	-	-
**pressure ratio**	0.5845	-	-
**peak position**	0.3196	-	-
**COP position**	0.2059	-	-

Table S3. Results of the statistical tests (paired T-test or Wilcoxon signed rank as post-hoc 2-way Anova or Friedman where appropriate) on the comparisons among the different hands positions (top handlebar – TH – hands on lever – LH – and drop handlebar – DH) in the whole saddle (WS), anterior saddle (AS) and posterior saddle (PS) for the conditions without and with the Equistasi ® device (noE and E respectively).

**Z or T values**	**WS**					
	**noE**			**E**		
	**TH vs LH**	**TH vs DH**	**LH vs DH**	**TH vs LH**	**TH vs DH**	**LH vs DH**
**average pressure**	0.0381	2.5814	3.6113	0.2101	4.2265	4.7414
**peak of pressure**	0.2594	2.9847	3.3331	0.2553	3.9680	4.7035
**contact surface**	-3.1364	2.3072	4.8953	-0.9563	4.4004	5.8114
**average force**	-1.8559	2.4652	3.8619	-0.6280	1.5792	5.8115
**pti**	0.0602	3.7450	4.1998	1.5983	4.3937	1.8216
**contact surface %**	-3.0609	2.4362	4.9473	-1.0932	4.5601	6.0468
**pressure ratio**	-1.1276	-1.6174	-0.4749	-1.3088	-2.4933	-1.2764
**peak position**	-1.1200	-2.5577	-1.5259	-0.9983	-2.6453	-1.7004
**COP position**	-1.0533	-2.2569	-1.2571	-0.9149	-2.3704	-1.5041

**Z or T values**	**AS**					
	**noE**			**E**		
	**TH vs LH**	**TH vs DH**	**LH vs DH**	**TH vs LH**	**TH vs DH**	**LH vs DH**
**average pressure**	-3.8646	-1.4719	0.8756	-2.8049	-1.2247	1.7962
**peak of pressure**	-3.9128	-1.6841	0.6368	-3.1160	-0.8043	1.5584
**contact surface**	-6.0659	-5.4266	-2.3631	-5.7175	-4.8832	-2.2387
**average force**	-5.2373	-4.7996	-1.4975	-1.0074	-3.3449	-0.3427

**Z or T values**	**PS**					
	**noE**			**E**		
	**TH vs LH**	**TH vs DH**	**LH vs DH**	**TH vs LH**	**TH vs DH**	**LH vs DH**
**average pressure**	0.5574	4.5863	4.9691	0.7984	3.0840	2.3001
**peak of pressure**	2.1889	2.3325	4.8174	0.9754	3.2496	2.3731
**contact surface**	2.9986	5.7028	5.6480	0.6930	6.4267	6.5939
**average force**	3.1421	5.1211	5.0149	3.1307	5.7928	6.0271

**p values**	**WS**					
	**noE**			**E**		
	**TH vs LH**	**TH vs DH**	**LH vs DH**	**TH vs LH**	**TH vs DH**	**LH vs DH**
**average pressure**	0.9697	0.0098	0.0003	0.8339	0.0000	0.0000
**peak of pressure**	0.7954	0.0028	0.0009	0.7989	0.0001	0.0000
**contact surface**	0.0017	0.0210	0.0000	0.3389	0.0000	0.0000

**average force**	0.0635	0.0137	0.0001	0.5300	0.1174	0.0000
**pti**	0.9520	0.0002	0.0000	0.1100	0.0000	0.0709
**contact surface %**	0.0022	0.0148	0.0000	0.2743	0.0000	0.0000
**pressure ratio**	0.2621	0.1088	0.6359	0.1934	0.0142	0.2046
**peak position**	0.2648	0.0117	0.1295	0.3200	0.0092	0.0915
**COP position**	0.2941	0.0257	0.2109	0.3619	0.0192	0.1349

**p values**	**AS**					
	**noE**			**E**		
	**TH vs LH**	**TH vs DH**	**LH vs DH**	**TH vs LH**	**TH vs DH**	**LH vs DH**
**average pressure**	0.0001	0.1411	0.3813	0.0050	0.2207	0.0725
**peak of pressure**	0.0001	0.0922	0.5243	0.0018	0.4212	0.1191
**contact surface**	0.0000	0.0000	0.0181	0.0000	0.0000	0.0252
**average force**	0.0000	0.0000	0.1343	0.3162	0.0008	0.7318

**p values**	**PS**					
	**noE**			**E**		
	**TH vs LH**	**TH vs DH**	**LH vs DH**	**TH vs LH**	**TH vs DH**	**LH vs DH**
**average pressure**	0.5782	0.0000	0.0000	0.4262	0.0025	0.0231
**peak of pressure**	0.0286	0.0215	0.0000	0.3314	0.0015	0.0193
**contact surface**	0.0027	0.0000	0.0000	0.4896	0.0000	0.0000
**average force**	0.0017	0.0000	0.0000	0.0017	0.0000	0.0000

Table S4. Results of the statistical tests (paired T-test or Wilcoxon signed rank as post-hoc 2-way Anova or Friedman where appropriate) on the comparisons among the different conditions without and with the Equistasi ® device (noE and E respectively) for the hands positions (top handlebar – TH – hands on lever – LH – and drop handlebar – DH) in the whole saddle (WS), anterior saddle (AS) and posterior saddle (PS).

**Z or T values**	**WS**		
	**noE vs E**		
	**TH**	**LH**	**DH**
**average pressure**	-1.3646	-1.2311	-2.3741
**peak of pressure**	-4.4325	-1.8835	-2.6827
**contact surface**	-5.5974	-5.7579	-4.7348
**average force**	-5.2585	-5.6335	-4.1337
**pti**	-3.8854	-4.0861	-2.5212
**contact surface %**	-5.8281	-5.9905	-4.9036
**pressure ratio**	1.0424	0.9390	0.1041
**peak position**	0.5894	0.7314	0.4451
**COP position**	0.7144	0.8959	0.6057

**Z or T values**	**AS**		
	**noE vs E**		
	**TH**	**LH**	**DH**
**average pressure**	-2.4293	-2.1512	-1.5386
**peak of pressure**	-1.9577	-2.2119	-0.9635
**contact surface**	-2.1138	-2.3236	-1.7633
**average force**	-2.0740	-2.4421	-1.4175

Z or T values	PS		
	noE vs E		
	TH	LH	DH
average pressure	-1.7918	-1.5772	-2.5324
peak of pressure	-1.8841	-4.8108	-0.8857
contact surface	-4.8217	-5.1093	-3.8596
average force	-4.5461	-5.1835	-3.9640

p values	WS		
	noE vs E		
	TH	LH	DH
average pressure	0.1748	0.2206	0.0176
peak of pressure	0.0000	0.0622	0.0073
contact surface	0.0000	0.0000	0.0000
average force	0.0000	0.0000	0.0000
pti	0.0001	0.0000	0.0117
contact surface %	0.0000	0.0000	0.0000
pressure ratio	0.2995	0.3499	0.9173
peak position	0.5566	0.4658	0.6570
COP position	0.4763	0.3719	0.5458

p values	AS		
	noE vs E		
	TH	LH	DH
average pressure	0.0151	0.0315	0.1239
peak of pressure	0.0503	0.0270	0.3353
contact surface	0.0345	0.0201	0.0779
average force	0.0381	0.0146	0.1563

p values	PS		
	noE vs E		
	TH	LH	DH
average pressure	0.0756	0.1173	0.0113
peak of pressure	0.0621	0.0000	0.3777
contact surface	0.0000	0.0000	0.0001
average force	0.0000	0.0000	0.0001

Supplementary material C

Results concerning the force in peak_E and the surface in peak_E

Results concerning the force in peak_E and the surface in peak_E were reported in Figures C1 and C2, and following tables. A statistically significant increase in the PS in all the positions can be noticed in both variables for the condition with Equistasi® (p<0.025).

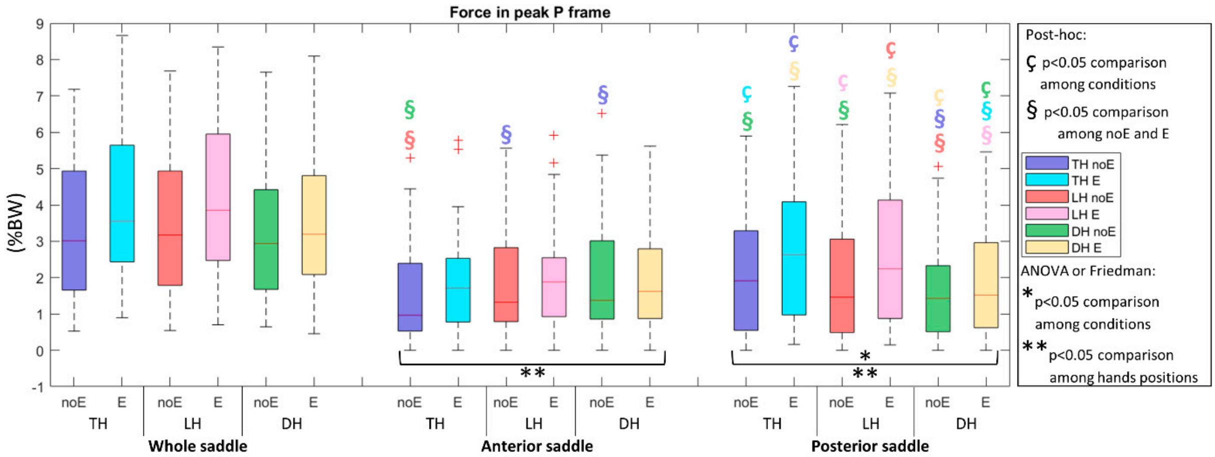

Figure S2. Force value in the frame corresponding to the pressure's peak in the Whole saddle, Anterior saddle and Posterior saddle when cycling in top handlebar (TH), hands on lever (LH) and drop handlebar (DH) positions, in the conditions without (noE) and with Equistasi® (E). * and ** means a statistically significant differences (p<0.05) with 2way-Anova or Friedman test in the comparison among conditions (*) or hands positions (**). ç and § means a statistically significant difference (p<0.05) with post-hoc test in the comparison among conditions (ç) or hands positions (§): blue with respect to TH noE, light blue with respect to TH E, red with respect to LH noE, pink with respect to LH E, green with respect to DH noE, yellow with respect to DH E.

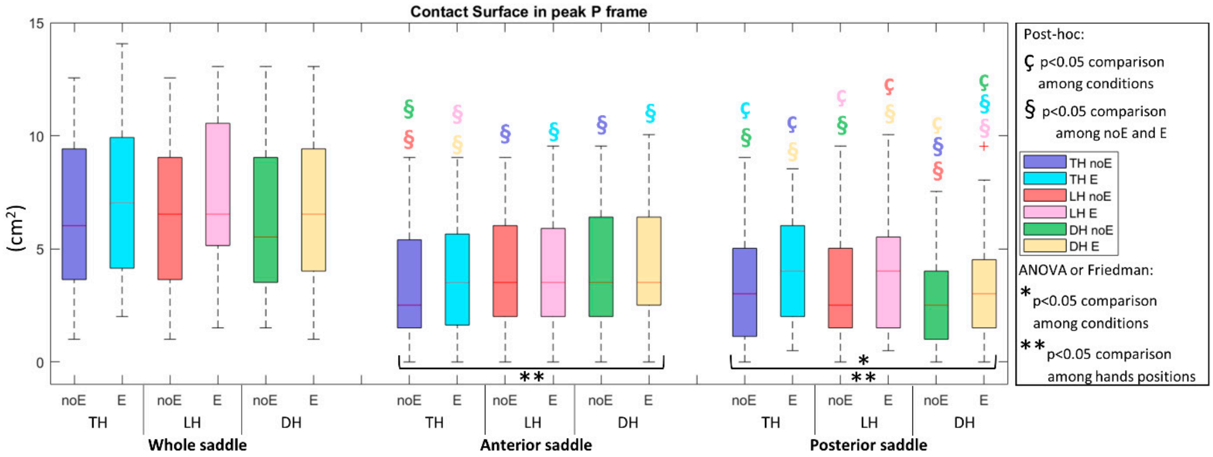

Figure S3. Contact surface in the frame corresponding to the peak of pressure in the Whole saddle, Anterior saddle and Posterior saddle when cycling with TH, LH and DH, in the conditions without (noE) and with Equistasi® (E). * and ** means a statistically significant differences (p<0.05) with 2way-Anova or Friedman test in the comparison among conditions (*) or hands positions (**). ç and § means a statistically significant difference (p<0.05) with post-hoc test in the comparison among conditions (ç) or hands positions (§): blue with respect to TH noE, light blue with respect to TH E, red with respect to LH noE, pink with respect to LH E, green with respect to DH noE, yellow with respect to DH E.

Table S5. Summary of the statistically significant differences on the comparisons among the different hands positions (top handlebar – TH – hands on lever – LH – and drop handlebar – DH) in the

whole saddle (WS), anterior saddle (AS) and posterior saddle (PS) for the conditions without and with the Equistasi ® device (noE and E respectively). p values are reported in brackets where significant differences occur (paired T-test or Wilcoxon signed rank as post-hoc 2-way Anova or Friedman where appropriate).

	TH vs LH		TH vs DH		LH vs DH	
	**noE**	**E**	**noE**	**E**	**noE**	**E**
**WS - force in peak**						
**WS - contact surface in peak**						
**AS - force in peak**	higher in LH (<0.001)		higher in DH (<0.001)			
**AS - contact surface in peak**	higher in LH (<0.001)	higher in LH (0.002)	higher in DH (<0.001)	higher in DH (<0.001)		
**PS - force in peak**			lower in DH (<0.001)		lower in DH (<0.001)	
**PS - contact surface in peak**			lower in DH (0.002)	lower in DH (0.014)	lower in DH (<0.001)	lower in DH (<0.001)

Table S6. Results of the statistical tests (2-way Anova or Friedman where appropriate) on the comparisons among the different hands positions without the Equistasi® device (top handlebar – TH – hands on lever – LH – and drop handlebar – DH) in the whole saddle (WS), anterior saddle (AS) and posterior saddle (PS).

**X2 or F values**	**TH vs LH vs DH**		
	**WS**	**AS**	**PS**
**force in peak**	3.0929	3.1579	8.8679
**contact surface in peak**	1.3153	2.6704	8.0099
**p values**	**TH vs LH vs DH**		
	**WS**	**AS**	**PS**
**force in peak**	0.2130	0.2062	0.0119
**contact surface in peak**	0.5181	0.2631	0.0182

Table S7. Results of the statistical tests (2-way Anova or Friedman where appropriate) on the comparisons among the different conditions with the Equistasi ® device for the hands positions (top handlebar – TH – hands on lever – LH – and drop handlebar – DH) in the whole saddle (WS), anterior saddle (AS) and posterior saddle (PS).

**X2 or F values**	**TH vs LH vs DH**		
	**WS**	**AS**	**PS**
**force in peak**	5.8137	2.1338	5.8795
**contact surface in peak**	3.8603	0.2940	6.7884
**p values**	**TH vs LH vs DH**		
	**WS**	**AS**	**PS**
**force in peak**	0.0159	0.1441	0.0153
**contact surface in peak**	0.0494	0.5877	0.0092

Table S8. Results of the statistical tests (paired T-test or Wilcoxon signed rank as post-hoc 2-way Anova or Friedman where appropriate) on the comparisons among the different hands positions (top handlebar – TH – hands on lever – LH – and drop handlebar – DH) in the whole saddle (WS), anterior saddle (AS) and posterior saddle (PS) for the conditions without and with the Equistasi ® device (noE and E respectively).

Z or T values	WS					
	noE			E		
	TH vs LH	TH vs DH	LH vs DH	TH vs LH	TH vs DH	LH vs DH
force in peak	-0.4275	0.3627	0.8299	-0.0085	1.6628	1.7007
contact surface in peak	-1.8842	-0.5511	1.0753	-0.0782	3.4808	3.8681
Z or T values	AS					
	noE			E		
	TH vs LH	TH vs DH	LH vs DH	TH vs LH	TH vs DH	LH vs DH
force in peak	-4.4308	-4.9334	-1.4709	-0.7401	-1.9049	0.0193
contact surface in peak	-4.1022	-4.9292	-1.9257	-3.0786	-3.4890	-1.4383
Z or T values	PS					
	noE			E		
	TH vs LH	TH vs DH	LH vs DH	TH vs LH	TH vs DH	LH vs DH
force in peak	0.9750	3.6876	3.9916	1.9169	5.4554	5.0055
contact surface in peak	0.1783	3.0222	3.4968	1.9053	2.5043	3.9826

p values	WS					
	noE			E		
	TH vs LH	TH vs DH	LH vs DH	TH vs LH	TH vs DH	LH vs DH
force in peak	0.6700	0.7176	0.4086	0.9932	0.0995	0.0921
contact surface in peak	0.0595	0.5816	0.2822	0.9377	0.0005	0.0001
p values	AS					
	noE			E		
	TH vs LH	TH vs DH	LH vs DH	TH vs LH	TH vs DH	LH vs DH
force in peak	0.0000	0.0000	0.1413	0.4610	0.0568	0.9846
contact surface in peak	0.0000	0.0000	0.0541	0.0021	0.0005	0.1504
p values	PS					
	noE			E		
	TH vs LH	TH vs DH	LH vs DH	TH vs LH	TH vs DH	LH vs DH
force in peak	0.3296	0.0002	0.0001	0.0553	0.0000	0.0000
contact surface in peak	0.8585	0.0025	0.0005	0.0567	0.0136	0.0001

Table S9. Results of the statistical tests (paired T-test or Wilcoxon signed rank as post-hoc 2-way Anova or Friedman where appropriate) on the comparisons among the different conditions without and with the Equistasi ® device (noE and E respectively) for the hands positions (top handlebar – TH – hands on lever – LH – and drop handlebar – DH) in the whole saddle (WS), anterior saddle (AS) and posterior saddle (PS).

Z or T values	WS - noE vs E		
	TH	LH	DH
force in peak	-1.9713	-1.6418	-0.7728
contact surface in peak	-5.1737	-3.5456	-1.1830
pti	-3.8854	-4.0861	-2.5212
contact surface %	-5.8281	-5.9905	-4.9036
pressure ratio	1.0424	0.9390	0.1041
peak position	0.5894	0.7314	0.4451
COP position	0.7144	0.8959	0.6057
	AS - noE vs E		
	TH	LH	DH

**force in peak**	-2.5415	-1.7128	-0.9641
**contact surface in peak**	-1.5412	-0.9376	-0.2794
		**PS - noE vs E**	
	**TH**	**LH**	**DH**
**force in peak**	-4.2556	-3.9275	-2.4371
**contact surface in peak**	-4.4816	-3.3586	-2.2411

**p values**		**WS - noE vs E**	
	**TH**	**LH**	**DH**
**force in peak**	0.0515	0.1038	0.4414
**contact surface in peak**	0.0000	0.0004	0.2368
		**AS - noE vs E**	
	**TH**	**LH**	**DH**
**force in peak**	0.0110	0.0867	0.3350
**contact surface in peak**	0.1233	0.3485	0.7800
		**PS - noE vs E**	
	**TH**	**LH**	**DH**
**force in peak**	0.0000	0.0001	0.0148
**contact surface in peak**	0.0000	0.0008	0.0250
